# The Effect of α-Tocopherol on the Reduction of Inflammatory Processes and the Negative Effect of Acrylamide

**DOI:** 10.3390/molecules27030965

**Published:** 2022-01-31

**Authors:** Marta Kopańska, Marta Batoryna, Agnieszka Banaś-Ząbczyk, Joanna Błajda, Marcin W. Lis

**Affiliations:** 1 Department of Pathophysiology, Institute of Medical Sciences, University of Rzeszow, 35-959 Rzeszów, Poland; 2 Department of Animal Physiology and Toxicology, Pedagogical University in Cracow, 30-059 Cracow, Poland; m.batoryna@gmail.com; 3 Department of Biology, Institute of Medical Sciences, University of Rzeszow, Rejtana 16c, 35-959 Rzeszów, Poland; agnieszkabanas@o2.pl; 4 College of Medical Sciences, University of Rzeszow, Rejtana 16c, 35-959 Rzeszów, Poland; joanna.blajda@gmail.com; 5 Department of Zoology and Animal Welfare, Faculty of Animal Science, University of Agriculture in Cracow, 30-059 Cracow, Poland; rzlis@cyfronet.pl

**Keywords:** cholinergic system, inflammation, tocopherol

## Abstract

Our research aimed to show acrylamide’s influence on inflammatory processes, the oxidative stress it causes in the cholinergic system, and the possibility of reducing inflammation via supplementation with α-tocopherol. For this purpose, an in ovo model was used where the embryos were exposed to acrylamide, α-tocopherol and a cocktail of these substances. After 48 h of exposure, we collected brain samples and performed biochemical assays to examine the effect of the chosen substances on oxidative stress (malondialdehyde-MDA and reduced glutathione-GSH) and acetylcholinesterase activity (AChE). The results showed that acrylamide decreased AChE activity in the examined brain samples by about 25% in comparison to the control group, and this effect was decreased by administering α-tocopherol. The concentration of malondialdehyde significantly increased in the group given acrylamide, while, in the group with α-tocopherol, the observed concentration was lower in comparison to the control group. Moreover, a decrease in glutathione concentration was observed after the administration of acrylamide; however, the protective effect of α-tocopherol was only slightly visible in this case. In conclusion, α-tocopherol minimizes the harmful effects of acrylamide on AchE, and it can minimize the concentration of MDA.

## 1. Introduction

The harmful effect of acrylamide (ACR) has already been well documented, and since the 1990s, many studies have been carried out to analyze the carcinogenicity, toxicity and neurotoxicity of acrylamide. Our research aims to show the influence of acrylamide on inflammatory processes causing oxidative stress in the cholinergic system. In addition, our focus was on investigating the possibility of reducing ACR harmful effects via supplementation with α-tocopherol. 

Acrylamide is an organic compound with the formula C_3_H_5_NO; it is formed due to the reaction between amino acids and reducing sugars in the Maillard reaction. It is formed in significant amounts during the thermal treatment, frying or baking of carbohydrate-containing food products, particularly starches. According to WHO data, chips, crisps, coffee, cakes and biscuits as well as bread and other foods make up the largest share of the total acrylamide consumed [1,2]. ACR action in the organism leads to the oxidative stress, inflammation, apoptosis and damage to DNA [3]. 

ACR treatment causes an inflammatory response and biochemical alterations through increased mRNA expression levels of NFĸB, IFN-γ, IL-1β and TNF-α in the liver and brains of rats [4]. Activation of the inflammatory process may result from oxidative stress induced by ACR. Increased amount of free radicals can activate inflammatory pathways, e.g., by activating the NLRP3 inflammasome [5,6].

Acetylcholine (ACh) is best known as a neurotransmitter. ACh signaling in the neuronal cholinergic system has long been known to regulate numerous biological processes. Furthermore, ACh is produced by numerous non-neuronal cell types, and the non-neuronal cholinergic system, which includes immune system functions, cooperates in parallel with the neuronal system. T cells respond to infection by producing ACh, and this ability to produce ACh is dependent upon IL-21 signaling to the T cells. 

Furthermore, during infection, this immune-derived ACh is necessary for the T cells to migrate into infected tissues [7,8]. The immunomodulatory effects of the efferent vagus nerve also play a role in localized peripheral inflammation. Electrical stimulation of the distal vagus nerve inhibits the local inflammatory response in a standard rodent model of carrageenan-induced paw edema. Pretreatment with acetylcholine, localized within the site of inflammation also prevents the development of swelling [9]. The cholinergic anti-inflammatory pathway could modulate inflammation in real time [5,10].

To maintain the oxidation–reduction balance, the body has complex antioxidant systems in which superoxide dismutase, catalase and glutathione are involved. These systems can be supported by substances with the above-mentioned properties taken from outside, for example vitamins. One of the known effective anti-oxidant and anti-inflammatory agents is vitamin E and its derivatives [11]. It is present in eight derivative forms, and the group α-Tocopherol (α-TOH) is known to be the most active anti-oxidant. 

α-TOH acts as protector against H_2_O_2_-induced lipid peroxidation due to increased anti-oxidative enzyme systems, such as glutathione and catalase [12], reduces oxidative stress-induced apoptosis [13] and acts as a regulator of genes involved in lipid metabolism, homeostasis and inflammation [14]. Although people consume relatively more of type γ, type α is predominant. This is due to the higher affinity of the transport protein (α-TTP). This helps transport type α to cell membranes in the body [15,16,17].

## 2. Results

### 2.1. The Influence of Tocopherol on Acetylcholinesterase Activity

The AChE activity was effected by ACR (*p* < 0.001) and Tocopherol dose (*p* = 0.003), but not brain area (*p* = 0.126). Moreover, we did not find a statistically significant interaction between: ACR and T dose (*p* = 0.729); ACR and the brain area (*p* = 0.906); or T dose and brain area (*p* = 0.962).

**Figure 1:**
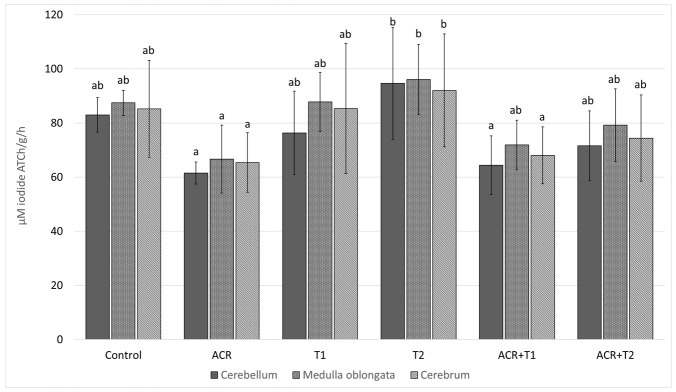
The activity of acetylcholinesterase (AChE, mean ± SD) in the selected brain areas of chicken embryos (19th day of incubation) treated in ovo with acrylamide (ACR, 2.4 mg per egg) and/or α-tocopherol (T1, 0.5 mg per egg; or T2, 5.0 mg per egg). The in ovo injection was performed at the 17th day of incubation. ab–values marked by various letters differ significantly (*p* < 0.05).

The AChE activity in the cerebellum, medulla oblongata and cerebrum of the control was (mean ± SD) 83.0 ± 6.37; 87.4 ± 4.70 and 85.2 ± 17.88 [μM iodide ATCh/g/h], respectively; Figure 1. In comparison to the control, ACR decreased in the particular brain areas by 25.9 (*p* = 0.154); 23.7% (*p* = 0.225) and 23.3% (*p* = 0.183). Concomitant ACR administration with T1 or T2 did not prevent this decline.

### 2.2. The Influence of Tocopherol on Malondialdehyde

The MDA concentration was effected by ACR (*p* < 0.001), T dose (*p*< 0.001), and a brain area (*p* = 0.046). Moreover, we found a statistically significant interaction between: ACR and T dose (*p* = 0.001); but not between the brain area and ACR (*p* = 0.127) and T dose (*p* = 0.164).

**Figure 2:**
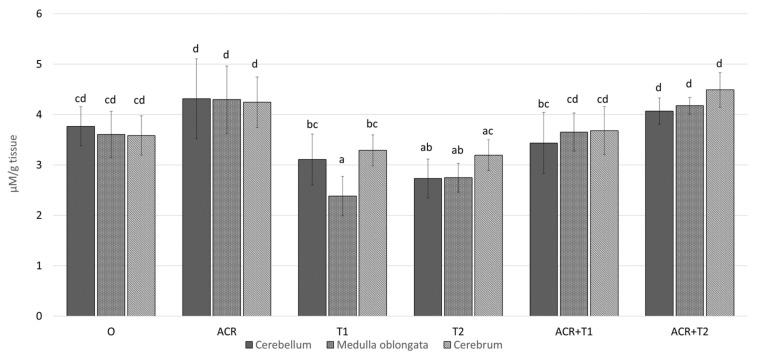
The concentration of malondialdehyde (MDA, mean ± SD) in the selected brain areas of chicken embryos (19th day of incubation) treated in ovo with acrylamide (ACR, 2.4 mg per egg) and/or α-tocopherol (T1, 0.5 mg per egg; or T2, 5.0 mg per egg). The in ovo injection was performed at the 17th day of incubation. abcd–values marked by various letters differ significantly (*p* < 0.05).

The MDA concentration in the cerebellum, medulla oblongata and cerebrum of the control was (mean ± SD) 3.8 ± 0.39; 3.6 ± 0.46 and 3.6 ± 0.39 [μM/g tissue], respectively; Figure 2. In comparison to the control, ACR slightly increased in the particular brain areas by 14.5 (*p* = 0.258); 19.2% (*p* = 0.073) and 18.4% (*p* = 0.159). On the other hand, the MDA concentration in T1 and T2 was lower by 1.2- (*p* <0.099) and 1.4-fold (*p* = 0.001) in the cerebellum; 1.5- (*p* < 0.001) and 1.3-fold (*p* = 0.008) in the medulla, and 1.1- (*p* < 0.667) and 1.1-fold (*p* = 0.888) in the cerebrum, respectively. Concomitant ACR administration with T1 or T2 raised this parameter in both brain areas to or slightly above level of the control.

### 2.3. The Influence of Tocopherol on the Analyzed Glutathione

The GSH concentration was effected by ACR (*p* = 0.001) and T dose (*p* < 0.001), but not brain area (*p* = 0.129). Moreover, we found a statistically significant interaction between T dose and ACR (*p* < 0.001) and the brain area (*p* = 0.001); but not between ACR and the brain area (*p* = 0.158).

**Figure 3:**
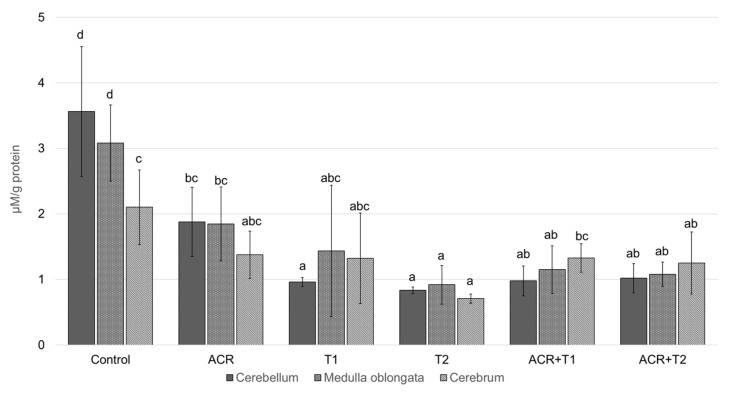
The concentration of glutathione (GSH, mean ± SD) in the selected brain areas of chicken embryos (19th day of incubation) treated in ovo with acrylamide (ACR, 2.4 mg per egg) and/or α-tocopherol (T1, 0.5 mg per egg; or T2, 5.0 mg per egg). The in ovo injection was performed at the 17th day of incubation. abcd–values marked by various letters differ significantly (*p* < 0.05).

The GSH concentration in the cerebellum, medulla oblongata and cerebrum of the control was (mean ± SD) 3.6 ± 0.99; 3.1 ± 0.58 and 3.1 ± 0.55 [μM/g protein], respectively Figure 3 and was significantly decreased by ACR—both doses of α-tocopherol and combinations of their substances (*p* < 0.001). The higher dose of tocopherol (T2) decreased the concentration of this antioxidant the most strongly. In comparison to the control and ACR, its value was lower 4,3- (*p* < 0.001) and 2,3-fold (*p* = 0.02) in the cerebellum; 3,4- (*p* < 0.001) and 2,0-fold (*p* = 0.057) in the medulla, and 4,4- (*p* < 0.001) 1,9-fold (*p* = 0.231) in the cerebrum, respectively. Concomitant ACR administration with T1 or T2 did not prevent this decline.

## 3. Discussion

In this article, we assessed the influence of acrylamide on oxidative stress, which may be the cause of inflammatory processes, as well as the influence of α-tocopherol on limiting its activity. Based on the examined factors, we can conclude that acrylamide disrupts the work of cholinergic system components. One of the factors tested was acetylcholinesterase (AChE). This is an enzyme whose task is to break down acetylcholine into choline and acetic acid [18]. Its presence is characteristic of the synapses of cholinergic neurons. These neurons have functional abilities in the brain and are assigned emotional, stress and memory functionalities. 

In addition, they are found in the motor plates of muscle fibers, due to which they influence control. Our results showed that, after ACR administration, the activity of AChE was decreased. Despite the lack of statistically significant differences after the administration of tocopherol with ACR in comparison to the ACR alone, there was a visible trend (especially in the higher dose ACR + T2) suggesting a reduction the harmful effects of ACR. This was confirmed by the fact that acrylamide may disturb synaptic transmission in cholinergic neurons of the central and peripheral nervous system by influencing the activity of AChE. 

Thus, ACR neurotoxicity can cause severe disturbances of synaptic transmission in cholinergic neurons in both the central and peripheral nervous systems. It is also known that the inhibition of AChE activity can cause overstimulation of postsynaptic membranes by acetylcholine, leading to the impairment of neurotransmission and muscle paralysis. 

This confirms the correlation of the effect of vitamin E on the improvement of the functionality of the cholinergic system when disturbed by inflammation. It is also possible that the effect of acrylamide on AChE activity may be due to a direct neurotoxic effect of the ACR or perhaps a disarrangement of the plasmatic membrane caused by increased lipid peroxidation. This is more likely when taking into consideration the MDA results and conclusions from another study [19].

As one study conducted on humans proved, degenerative changes lead to an increase in oxidative stress and thus an increase in the frequency of infections, inflammation and immune reactions. Studies have shown that supplementation with αTOH increases the immune response [20]. Our study confirmed that the same compound also supports anti-inflammatory processes in the cholinergic system. In addition to the cholinergic system, other authors argue that αTOH reduces oxidative stress in other systems. 

In addition, it can lead to a reduction in plasma lipid peroxides, thus, leading to an increase in the production of IL-2 in lymphocytes, which were additionally stimulated with Con A. The ability of tocopherol to lower lipid peroxidation is clearly visible in our results on the level of malondialdehyde (MDA). Different brain areas examined in the present study show different susceptibility to ACR and tocopherol. Similar conclusion can be found in other studies [19,21]

The differences in the level of reduced glutathione (GSH) in groups may result from two methods of acrylamide action and different activities of GSH. Due to the free thiol group, GSH is used to reduce peroxides (e.g., hydrogen peroxide). This captures reactive electrophilic factors, protecting cells against damage from toxins. This may explain why tocopherol alone led to a decrease in GSH levels, assuming that tocopherol took over the peroxide reductions and that the orgasm did not need more glutathione. Moreover, acrylamide has the ability to combine with glutathione [22], and thus, at the same time, it causes the formation of free radicals but also directly affects its reduction. In addition, the increase in the ability of alpha-tocopherol to inhibit nuclear lipid peroxidation by glutathione could also lead to a decrease in GSH levels [23].

Research also proved [24] that αTOH does not always result in positive immunological phenomena. In the case of patients with periodic allergic rhinitis, it did not affect the general phenomena and IgE level but only slightly improved some parameters. A more detailed analysis should be performed and the therapeutic efficacy compared with other doses and research protocols. In addition, in αTOH studies, when used in the treatment of cardiological and nephrological patients, people with diabetes and in healthy people, it helped improve the system’s parameters [25,26]. 

Vitamin E has also been used in the prevention of osteoporosis [27]. Many studies have also sought to establish the protective antioxidant role of vitamin E regarding asthma [24]. Our research shows the need to deepen knowledge and continue research into the neurological aspect as we assume that the use of αTOH may be an effective therapeutic method in treating some dysfunctions of the human cholinergic system.

Our research was not conducted on human material; however, as other studies have shown, there are robust premises as to the possibility of supporting our hypothesis.

## 4. Materials and Methods

Hatching eggs (*n* = 90, weight (mean ± SD) 62.7 ± 6.11 g) of Ross 308 broiler chicken parental flock (Aviagen) were obtained from a commercial farm (Sławomir Domagała, Gołaczewy, Poland) and incubated in a Ova-Easy 380 incubator (Brinsea, Titusville, FL, USA) at a temperature of 37.8 ± 0.1 °C and a relative humidity of 50% ± 2%. On the 7th and 17th days of incubation (E7 and E17), the eggs were candled with an ovoscope to determine embryo development, and infertile and dead embryo eggs were rejected. The embryonated eggs were randomly divided into six groups (*n* = 10 eggs per group) and subjected to *in ovo* injection [17,18]. 

A hole (1.2 mm diameter) was aseptically drilled in the egg shell’s air cell region using a G18 needle. The eggs were administered simultaneously with acrylamide (Merck KGaA, Darmstadt, German) at a dose of 0.0 or 2.4 mg dissolved in 0.1 mL 0.7% NaCl and α-tocopherol (Merck KGaA, Darmstadt, German) at a dose of 0.0, 0.5 or 5.0 mg dissolved in 0.1 mL archid oil (Merck KGaA, Darmstadt, German) (Table 1) using BD 1-mL conventional insulin syringes (BD Switzerland Sarl, Eysins, Switzerland) and G25 needles into the albumen under the chorioallantoic membrane. Next, the hole was sealed with hot wax, and incubation was continued.

The embryos were euthanized by decapitation on E19 (i.e., after 48 h of experimental exposure) even ahead of the natural increase level of oxidative stress during the internal pipping [28,29], and the brain tissue samples (cerebrum, cerebellum, medulla oblongata) were collected, immediately frozen and stored at −80 °C until further analysis.

The general protein concentration was determined using Bradford’s method [30]. The calibration curve was prepared with bovine albumin (BSA) in the range of protein concentration of 0.1–1.4 mg/mL. The measurement was performed by using the microplate reader SUNRISE™ (TECAN, Switzerland) at a wavelength λ = 595 nm.

The malondialdehyde (MDA) concentration was determined, according to Ohkawa based on the double-heating method reaction of thiobarbituric acid (TBA) with MDA [31]. The tissues (1 mg) were homogenized in the homogenizer (CAT X360) with 10 mL of a RIPA buffer (cell lysis solution reagent) containing 0.1% v/v protease inhibitor. After centrifugation (4500 rpm), sodium dodecyl sulfate (SDS), trichloroacetic acid (TCA), and TBA were added to the sample and boiled. Next, the sample was cooled and centrifuged, and the supernatant was placed on the microplate reader SUNRISE™ (TECAN, Switzerland) to measure the absorbance at λ = 535 nm with respect to the blank solution. The results are presented as μM/mg tissue.

The reduced glutathione (GSH) concentration was measured with Ellman’s method [32]. The tissue homogenates were prepared in a phosphate buffer with EDTA, pH 7.4. The GSH concentrations were measured at the absorbance of λ = 412 nm in deproteinised supernatants using the microplate reader SUNRISE™ (TECAN, Switzerland).

For AChE measurements, brain tissues were homogenized in an ice cooled (4 °C) 0.1 M sodium phosphate buffer (pH = 8) in the proportion of 1 mL buffer/20 mg of the tissue. After centrifugation, a supernatant was used to measure acetylcholinesterase via Ellman’s colorimetric procedures [32]. The method is based on the hydrolysis of acetylthiocholine or butyryltiocholine iodide, where the final product is thiocholine. 

Thiocholine in the presence of the highly reactive dithiobis-2-nitro-benzoic acid (DTNB) reacts to generate the yellow 5-thio-2-nitrobenzoate product. Absorbance was measured at λ = 412 nm after 60 s. The results are presented as μM of acetylthiocholine iodide per g tissue/h. Detection was performed with a MARCEL S330 spectrophotometer.

### Statistical Analyses

The effect of factors (i.e., the acrylamide and tocopherol dose and brain area) on AChE activity and GSH and MDA concentration were analyzed by a three-way ANOVA, followed by Pairwise Multiple Comparison Procedures by Holm–Sidak method (*post hoc* test). The overall significance level was = 0.05. The results were presented as the means ± SD. The statistical procedures were performed using Sigma-Stat 3.5 (Systat Software Inc., Palo Alto, CA, USA).

## 5. Conclusions

1. Relationships between exposure to acrylamide an a decrease in AChE and GSH activity were demonstrated.
2. α-Tocopherol reduces the harmful effects of acrylamide on AChE.
3. Relationships between exposure to acrylamide and an increase in MDA, one of the markers of oxidative stress, were demonstrated.
4. α-Tocopherol can reduce MDA concentration.
5. α-Tocopherol can reduce the toxic effects of acrylamide, thus increasing the level of GSH.
6. Acrylamide is a substance that negatively affects the cholinergic system.

## Figures and Tables

**Table 1:** Schema of experimental groups and the dosage of experimental substances (dose per egg). The in ovo injection was performed on the 17th day of incubation.

Group	α-Tocopherol	Dissolvent to α-Tocopherol (Archid Oil)	Acrylamide	Dissolvent to Acrylamide (0.7% NaCl)
C (control)	0.0 mg	0.1 mL	0.0 mg	0.1 mL
ACR	0.0 mg	0.1 mL	2.4 mg	0.1 mL
T1	0.5 mg	0.1 mL	0.0 mg	0.1 mL
T2	5.0 mg	0.1 mL	0.0 mg	0.1 mL
ACR + T1	0.5 mg	0.1 mL	2.4 mg	0.1 mL
ACR + T2	5.0 mg	0.1 mL	2.4 mg	0.1 mL

## Data Availability

The data presented in this study are available on request from the corresponding author.

## Sample Availability

Samples of the compounds are available from the authors.

## References

[1] FAO, WHO (2005). Evaluation of Certain Food Contaminants-Sixty-Fourth Report of the Joint FAO/WHO Expert Committee on Food Additives.

[2] Rice J.M. (2005). The carcinogenicity of acrylamide. Mutat. Res.-Genet. Toxicol. Environ. Mutagen..

[3] Sengul E., Gelen V., Yildirim S., Tekin S., Dag Y. (2020). The Effects of Selenium in Acrylamide-Induced Nephrotoxicity in Rats: Roles of Oxidative Stress, Inflammation, Apoptosis, and DNA Damage. Biol. Trace Element Res..

[4] Zeki A., Ince S., Arslan-Acaroz D., Gurler Z., Kucukkurt I., Demirel H.H., Arslan H.O., Varol N., Zhu K. (2018). The ameliorative effects of boron against acrylamide-induced oxidative stress, inflammatory response, and metabolic changes in rats. Food Chem. Toxicol..

[5] Bo N., Yilin H., Chaoyue Y., Lu L., Yuan Y. (2020). Acrylamide induces NLRP3 inflammasome activation via oxidative stress- and endoplasmic reticulum stress-mediated MAPK pathway in HepG2 cells. Food Che. Toxicol..

[6] Amirshahrokhi K. (2021). Acrylamide exposure aggravates the development of ulcerative colitis in mice through activation of NF-κB, inflammatory cytokines, iNOS, and oxidative stress. Iran. J. Basic Med. Sci..

[7] Fujii T., Mashimo M., Moriwaki Y., Misawa H., Ono S., Horiguchi K., Kawashima K. (2017). Expression and Function of the Cholinergic System in Immune Cells. Front. Immunol..

[8] Cox M.A., Bassi C., Saunders M.E., Nechanitzky R., Morgado-Palacin I., Zheng C., Mak T.W. (2019). Beyond neurotransmission: Acetylcholine in immunity and inflammation. J. Intern. Med..

[9] Borovikova L.V., Ivanovaa S., Nardia D., Zhanga M., Yanga H., Ombrellinob M., Tracey K.J. (2000). Role of vagus nerve signaling in CNI-1493-mediated suppression of acute inflammation. Auton. Neurosci..

[10] Han B., Li X., Hao J. (2017). The cholinergic anti-inflammatory pathway: An innovative treatment strategy for neurological diseases. Neurosci. Biobehav. Rev..

[11] Wallert M., Ziegler M., Wang X., Maluenda A., Xu X., Yap M.L., Witt R., Giles C., Kluge S., Hortmann M. (2019). α-Tocopherol preserves cardiac function by reducing oxidative stress and inflammation in ischemia/reperfusion injury. Redox Biol..

[12] Nakamura Y.K., Omaye S.T. (2008). α-Tocopherol modulates human umbilical vein endothelial cell expression of Cu/Zn superoxide dismutase and catalase and lipid peroxidation. Nutr. Res..

[13] Uemura M., Manabe H., Yoshida N., Fujita N., Ochiai J., Matsumoto N., Takagi T., Naito Y., Yoshikawa T. (2002). Alpha-tocopherol prevents apoptosis of vascular endothelial cells via a mechanism exceeding that of mere antioxidation. Eur. J. Pharmacol..

[14] Wallert M., Schmölz L., Galli F., Birringer M., Lorkowski S. (2014). Regulatory metabolites of vitamin E and their putative relevance for atherogenesis. Redox Biol..

[15] Lee G.Y., Han S.N. (2018). The Role of Vitamin E in Immunity. Nutrients.

[16] Traber M.G. (2007). Vitamin E Regulatory Mechanisms. Annu. Rev. Nutr..

[17] Ball G.F.M. (1998). Vitamin E. Bioavailability and Analysis of Vitamins in Foods.

[18] Voet D., Voet J.G., Pratt C.W. (2008). Fundamentals of Biochemistry: Life at the Molecular Level.

[19] Rosilene R.R., Corrêa M.C., Spanevello R.M., Morsch V.M., Mazzanti C.M., Gonçalves J.F., Schetinger M.R. (2005). Acetylcholinesterase activation and enhanced lipid peroxidation after long-term exposure to low levels of aluminum on different mouse brain regions. J. Inorg. Biochem..

[20] Wallert M., Börmel L., Lorkowski S. (2021). Inflammatory Diseases and Vitamin E—What Do We Know and Where Do We Go?. Mol. Nutr. Food Res..

[21] Bano S., Parihar M.S. (1997). Reduction of lipid peroxidation in different brain regions by a combination of α-tocopherol and ascorbic acid. J. Neural Transm..

[22] Zhu Y., Luo Y., Sun G., Wang P., Hu X., Chen F. (2020). Role of glutathione on acrylamide inhibition: Transformation products and mechanism. Food Chem..

[23] Tirmenstein M., Reed D.J. (1989). Effects of glutathione on the alpha-tocopherol-dependent inhibition of nuclear lipid peroxidation. J. Lipid Res..

[24] Abdala-Valencia H., Berdnikovs S., Cook-Mills J.M. (2013). Vitamin E Isoforms as Modulators of Lung Inflammation. Nutrients.

[25] Devaraj S., Tang R., Adams-Huet B., Harris A., Seenivasan T., De Lemos J.A., Jialal I. (2007). Effect of high-dose α-tocopherol supplementation on biomarkers of oxidative stress and inflammation and carotid atherosclerosis in patients with coronary artery diseasew. Am. J. Clin. Nutr..

[26] Nazrun A.S., Norazlina M., Norliza M., Nirwana S.I. (2011). The Anti-Inflammatory Role of Vitamin E in Prevention of Osteoporosis. Adv. Pharmacol. Sci..

[27] Reiter E., Jiang Q., Christen S. (2007). Anti-inflammatory properties of α-and γ-tocopherol. Mol. Asp. Med..

[28] Batoryna M., Lis M., Formicki G. (2017). Acrylamide-induced disturbance of the redox balance in the chick embryonic brain. J. Environ. Sci. Health Part B.

[29] Lis M.W., Głodek K., Sechman A., Wator D., Pawlak K., Niedziółka J.W. (2011). Effect of in ovo injection of aroclor 1254 on embryonic development, time of hatching, and blood thyroid hormone levels in one-day-old chicken. Bull. Vet. Inst. Pulawy.

[30] Bradford M.M. (1976). A rapid and sensitive method for the quantitation of microgram quantities of protein utilizing the principle of protein-dye binding. Anal. Biochem..

[31] Ohkawa H., Ohishi N., Yagi K. (1979). Assay for lipid peroxides in animal tissues by thiobarbituric acid reaction. Anal. Biochem..

[32] Ellman G.L., Courtney K.D., Andres V., Featherstone R.M. (1961). A new and rapid colorimetric determination of acetylcholinesterase activity. Biochem. Pharmacol..

